# Mature‐onset obesity in p62‐deficient male mice maintains skeletal muscle mass despite metabolic dysfunction

**DOI:** 10.14814/phy2.70851

**Published:** 2026-04-14

**Authors:** Do‐Houn Kim, Gloria Salazar, Alex Klemp, Hyun‐Seok Hwang, Mingchia Yeh, Lynn B. Panton, Jeong‐Su Kim

**Affiliations:** ^1^ Department of Health, Nutrition, and Food Sciences The Florida State University Tallahassee Florida USA; ^2^ Department of Biological and Health Sciences Texas A&M University‐Kingsville Kingsville Texas USA; ^3^ Department of Exercise Science Slippery Rock University Slippery Rock Pennsylvania USA; ^4^ Institute of Successful Longevity, The Florida State University Tallahassee Florida USA

**Keywords:** autophagy, insulin resistance, metabolic dysfunction, mTOR signaling, NBR1, neuromuscular health, obesity, sequestosome 1, skeletal muscle preservation

## Abstract

Sequestosome 1 (p62/SQSTM1) is a multifunctional scaffolding protein at the intersection of autophagy and metabolic regulation. While p62 deficiency causes mature‐onset obesity and insulin resistance, its effects on skeletal muscle mass and function remain poorly understood. Male p62 knockout (p62^−^/^−^) and wildtype control mice (*n* = 9/group) were studied from 14 to 34 weeks of age. p62^−^/^−^ mice exhibited greater food intake (+19.1%, *p* = 0.016), progressive weight gain (+41.5%, *p* < 0.001), and markedly elevated fat mass (+72.2%, *p* = 0.001), while lean mass was unchanged. Fasting hyperglycemia and impaired glucose tolerance (*p* < 0.05) confirmed systemic metabolic dysfunction. Despite this, p62^−^/^−^ mice maintained grip strength, skeletal muscle weights, and myofiber cross‐sectional areas comparable to controls. Molecular analysis revealed significantly elevated NBR1 (+61.6%, *p* = 0.020) and phospho‐mTOR (+78.2%, *p* = 0.033) in soleus muscle, suggesting altered autophagic flux. p62‐deficient male mice developed severe obesity and insulin resistance while maintaining skeletal muscle mass and grip strength at this intermediate timepoint. This phenotype was associated with altered mTOR and autophagy signaling. Whether muscle preservation is sustained long‐term warrants further investigation, as chronic obesity and metabolic dysfunction may ultimately impair muscle health.

## 1 INTRODUCTION

Obesity represents one of the most pressing global health challenges of the 21st century, characterized by excessive accumulation of adipose tissue that adversely affects virtually all physiological systems (World Health Organization, 2003). The pathophysiological consequences of obesity extend far beyond cosmetic concerns, encompassing increased risk for multiple chronic diseases including type 2 diabetes mellitus, cardiovascular disease, certain cancers, and neuromuscular disorders (World Health Organization, 2003). The complexity of obesity as a multifactorial condition involves intricate interactions between genetic predisposition, environmental factors, and behavioral patterns, particularly exposure to obesogenic environments characterized by high‐caloric diets and sedentary lifestyles.

Animal models that recapitulate key features of human obesity have proven invaluable in advancing our knowledge of metabolic disease pathogenesis and identifying potential therapeutic targets. These models provide controlled experimental platforms for investigating the complex interplay between genetic factors, metabolic dysfunction, and tissue‐specific responses to obesity‐induced stress.

Sequestosome 1, commonly referred to as p62 or SQSTM1, has emerged as a critical regulatory protein in cellular homeostasis and metabolic control (Bjørkøy et al., 2009). This multifunctional ubiquitin‐binding scaffolding protein serves as a crucial component of the autophagy machinery, functioning as a selective autophagy receptor that delivers ubiquitinated protein aggregates and damaged organelles to autophagosomes for degradation (Bjørkøy et al., 2009). The strategic positioning of p62 at the intersection of protein quality control, cellular stress responses, and metabolic signaling pathways has positioned it as a key player in maintaining cellular homeostasis under both physiological and pathological conditions.

Recent investigations have revealed that p62 extends its influence beyond autophagy regulation to encompass diverse metabolic processes including adipogenesis, inflammatory responses, and oxidative stress management (Diaz‐Meco & Moscat, 2012; Jiang et al., 2015; Lee et al., 2010). The development of p62‐deficient mouse models has provided valuable insights into the metabolic consequences of p62 loss. Pioneering studies demonstrated that mice lacking the p62 gene develop mature‐onset obesity accompanied by systemic glucose intolerance and insulin resistance (Hiruma et al., 2008; Rodriguez et al., 2006). These metabolic abnormalities were associated with impaired insulin‐induced insulin receptor substrate 1 (IRS‐1) activation in both adipose tissue and skeletal muscle, suggesting tissue‐specific mechanisms underlying the metabolic dysfunction. Subsequent investigations revealed that young adult p62‐deficient mice exhibit accelerated weight gain and enhanced adiposity development compared to wildtype controls (Harada et al., 2013). Importantly, these studies established that the insulin resistance observed in obese p62‐deficient mice was secondary to obesity development rather than a primary consequence of p62 deficiency, as metabolic defects were not apparent in young, lean p62‐deficient animals.

While previous studies have characterized the metabolic phenotype of p62‐deficient mice (Harada et al., 2013; Rodriguez et al., 2006), the effects on skeletal muscle mass, function, and molecular signaling have not been systematically investigated. Skeletal muscle represents the largest metabolically active tissue in the body and plays crucial roles in glucose homeostasis, protein metabolism, and overall metabolic health (Fatani et al., 2012; Friedman et al., 2012; Tamilselvan et al., 2013). Given the well‐established association between obesity and muscle dysfunction, including sarcopenia and dynapenia (Griffin et al., 2010; Park et al., 2007), and the critical role of autophagy in muscle homeostasis (Milan et al., 2015), understanding how p62 deficiency affects muscle biology in the context of obesity has important implications for both basic science and clinical translation.

The present investigation was designed to address these knowledge gaps by conducting a longitudinal evaluation of the physical and metabolic characteristics of p62‐deficient male mice from 14 to 34 weeks of age, with a particular emphasis on skeletal muscle biology. Specifically, we assessed neuromuscular function using grip strength and sensorimotor testing, characterized muscle morphology via histology, and examined the expression of key regulatory proteins involved in muscle protein turnover, glucose metabolism, and inflammation. We acknowledge that this study represents a cross‐sectional snapshot at an intermediate timepoint and does not include insulin‐stimulated conditions or direct measures of protein synthesis and degradation. Rather, our aim was to establish the baseline muscle phenotype of this model and identify molecular signatures that may inform future mechanistic investigations.

## 2 MATERIALS AND METHODS

### 2.1 Animal model and experimental design

Male C57BL/6 p62 knockout mice (p62^−^/^−^) and age‐matched wildtype controls were obtained at 14 weeks of age and studied until 34 weeks of age. The sample size of *n* = 9 per group was determined based on power analysis calculations using G*Power 3.1.9.7 software, assuming an effect size of 1.2 (based on preliminary data), *α* = 0.05, and power = 0.80 for detecting significant differences in primary outcome measures. This sample size provides adequate statistical power while adhering to the principles of animal welfare and the 3Rs (replacement, reduction, and refinement).

All experimental procedures were conducted in strict accordance with the “Principles of Laboratory Animal Care” (NIH publication No. 85‐23, revised 1985) and approved by the Animal Care and Use Committee of the Florida State University (Protocol number: 1703). Mice were individually housed in a pathogen‐free environment maintained at 22°C ± 2°C with a 12:12 h light–dark cycle. Animals had ad libitum access to standard rodent chow (Teklad Global 18% Protein Rodent Diet, Envigo, Indianapolis, IN) and filtered water throughout the study period.

The experimental timeline included biweekly assessments of body weight and food intake from 14 to 34 weeks of age. Comprehensive physiological evaluations were conducted at 24 and 34 weeks of age, including body composition analysis, neuromuscular function testing, and oral glucose tolerance tests. At study termination (34 weeks), animals were euthanized under deep anesthesia for tissue collection and subsequent molecular analyses.

### 2.2 Body composition analysis

Body composition was assessed using dual‐energy X‐ray absorptiometry (DXA) (iDXA Lunar, GE Healthcare, Madison, WI) equipped with small animal total body scan software. Prior to scanning, mice were anesthetized via subcutaneous injection of ketamine/xylazine (70 mg/kg and 3 mg/kg body weight, respectively). The DXA system was calibrated daily using manufacturer‐provided phantoms to ensure measurement accuracy and precision.

Outcome measures included total body mass, lean mass, fat mass, percent fat mass, and bone mineral density. All scans were performed by the same trained technician to minimize inter‐operator variability. The coefficient of variation for repeated measurements was <2% for all parameters, confirming the reliability of the assessment method (Khamoui et al., 2016).

### 2.3 Oral glucose tolerance test (OGTT)

Whole‐body glucose tolerance was evaluated using a standardized oral glucose tolerance test (OGTT) protocol. Following an overnight fast (>12 h), baseline fasting blood glucose (fBG) was measured using a lateral tail incision and the Bayer Contour glucose monitoring system (Bayer Consumer Care AG, Basel, Switzerland). The glucose meter was calibrated daily using manufacturer‐provided control solutions to ensure measurement accuracy.

Immediately after baseline blood collection, mice received 2 g/kg body weight of glucose solution (20% w/v in sterile water) via oral gavage. Subsequent blood glucose measurements were obtained at 15 (p15BG), 30 (p30BG), 60 (p60BG), and 120 min (p120BG) post‐glucose administration. Blood samples were collected from the same tail incision site or by making a fresh incision when necessary. The area under the curve (AUC) for glucose response was calculated using the trapezoidal rule to provide an integrated measure of glucose tolerance (Hoff, 2000).

### 2.4 Neuromuscular function assessment

#### 2.4.1 Inclined plane test

Sensorimotor function was evaluated using a standardized inclined plane test protocol. Mice were placed on a rectangular Plexiglas surface (60 × 122 cm) positioned at an initial angle of 30°. Success was defined as the ability to maintain position without sliding backward for five consecutive seconds. The angle was incrementally increased by 2° until the mouse failed to maintain position. Each mouse was allowed a maximum of three trials at each angle, with the highest successful angle recorded for analysis. This test provides a reliable measure of sensorimotor integration and muscle strength (Lee et al., 2015).

#### 2.4.2 Grip strength assessment

Forelimb grip strength was measured using a calibrated strain gauge (DFS‐101, AMETEK TCI, San Luis Obispo, CA). The device was calibrated before each testing session using known weights to ensure measurement accuracy. Mice were positioned so that their forelimbs grasped a horizontal tension bar, and they were gently pulled backward until grip was lost. The maximum force generated during the grip attempt was recorded in grams. Each mouse performed three trials with 30‐s rest intervals between attempts, and the highest force measurement was used for analysis (Kim et al., 2012).

### 2.5 Tissue collection and processing

At study termination, mice were anesthetized using 4.0%–4.5% isoflurane in medical‐grade oxygen and euthanized by cervical dislocation. The spleen and hindlimb flexor muscles (gastrocnemius, soleus, and plantaris) were rapidly excised from both hindlimbs. Right hindlimb muscles and spleen were immediately weighed on an analytical balance (precision ±0.1 mg), snap‐frozen in liquid nitrogen, and stored at −80°C for subsequent protein analysis. Left hindlimb muscles were prepared for histological analysis by mounting on cork bases using tragacanth gum and optical cutting temperature (OCT) compound, then frozen in liquid nitrogen‐cooled isopentane. This rapid freezing protocol preserves tissue morphology and prevents ice crystal formation that could compromise histological analysis.

### 2.6 Histological analysis and Myofiber cross‐sectional area

Transverse muscle sections (8–12 μm thickness) were cut from the mid‐belly region of the gastrocnemius muscle using a cryostat maintained at −20°C (Leica CM3050 S, Leica Biosystems, Buffalo Grove, IL). Sections were stained with hematoxylin and eosin using standard protocols to visualize muscle fiber morphology.

High‐resolution digital images were captured at 40× magnification using a light microscope equipped with a digital camera system (Olympus BX51, Olympus Corporation, Tokyo, Japan). Myofiber cross‐sectional area (CSA) was quantified using NIH ImageJ software (version 1.52) by a single investigator blinded to experimental group assignment. A minimum of 100 fibers per muscle section were analyzed, with the total area within the sarcolemma boundary used to determine CSA expressed in μm^2^. The coefficient of variation for repeated measurements was <5%, confirming the reliability of the quantification method (Khamoui et al., 2016).

### 2.7 Western blot analysis

#### 2.7.1 Protein extraction and quantification

Frozen muscle samples (approximately 50 mg) were homogenized in ice‐cold lysis buffer containing 50 mM HEPES (pH 7.4), 0.1% Triton X‐100, 4 mM EGTA, 10 mM EDTA, 50 mM sodium pyrophosphate tetrabasic, 100 mM β‐glycerophosphate, 25 mM sodium fluoride, 5 mM sodium metavanadate, and 10 μL/mL protease inhibitor cocktail (#8340, Sigma‐Aldrich, St. Louis, MO). Homogenization was performed using a tissue homogenizer (PowerGen 125, Fisher Scientific, Hampton, NH) with three 10‐s bursts at maximum speed, with 30‐s intervals on ice between bursts.

Homogenates were centrifuged at 12,000 × g for 15 min at 4°C, and the supernatant was collected for protein analysis. Protein concentrations were determined using the Bradford reagent (Bio‐Rad Protein Assay, Bio‐Rad Laboratories, Hercules, CA) with bovine serum albumin as the standard (Bradford, 1976). All samples were assayed in triplicate, and the coefficient of variation was <5% for all measurements.

#### 2.7.2 Gel electrophoresis and transfer

Equal amounts of protein (40 μg per lane) were mixed with 4× Laemmli sample buffer containing β‐mercaptoethanol and heated at 95°C for 5 min to denature proteins. Samples were separated on 4%–20% gradient SDS‐polyacrylamide precast gels (Mini‐PROTEAN TGX, Bio‐Rad Laboratories) using a Mini‐PROTEAN Tetra Cell system. Electrophoresis was performed at 120 V for approximately 90 min until the dye front reached the bottom of the gel. Proteins were transferred to polyvinylidene difluoride (PVDF) membranes (Immobilon‐P, Cat# IPVH00010, Millipore, Burlington, MA) using a Trans‐Blot Turbo Transfer System (Bio‐Rad Laboratories) at 25 V for 7 min. Transfer efficiency was verified using Ponceau S staining, and membranes were photographed for documentation of equal loading and transfer quality.

#### 2.7.3 Antibody incubation and detection

Membranes were blocked in 5% nonfat dry milk in Tris‐buffered saline with 0.1% Tween 20 (TBS‐T) for 1 h at room temperature with gentle agitation. Primary antibodies were diluted in blocking buffer and incubated overnight at 4°C with gentle rocking. The following primary antibodies were used: anti‐mTOR (#2972, Cell Signaling Technology, Danvers, MA), anti‐phospho‐mTOR (Ser2448) (#2971, Cell Signaling Technology), anti‐Akt (#9272, Cell Signaling Technology), anti‐phospho‐Akt (Ser473) (#9271, Cell Signaling Technology), anti‐MuRF1 (#33973, Cell Signaling Technology), anti‐Atrogin1/FBXO32 (12866‐1‐AP, Proteintech, Rosemont, IL), anti‐Insulin Receptor β (#3025, Cell Signaling Technology), anti‐GLUT4 (#2213, Cell Signaling Technology), anti‐TNF‐α (#3707, Cell Signaling Technology), anti‐NBR1 (#5202, Cell Signaling Technology), and anti‐GAPDH (#2118, Cell Signaling Technology) as loading control. All primary antibodies were used at 1:1000 dilution unless otherwise specified. After primary antibody incubation, membranes were washed three times for 10 min each in TBS‐T and incubated with appropriate horseradish peroxidase‐conjugated secondary antibodies (anti‐rabbit IgG #7074 or anti‐mouse IgG #7076, Cell Signaling Technology) at 1:10,000 dilution for 1 h at room temperature.

Following three additional washes in TBS‐T, protein bands were visualized using enhanced chemiluminescence substrate (West Pico PLUS, Cat# 34580, Thermo Fisher Scientific, Waltham, MA) and detected using a ChemiDoc XRS+ imaging system (Bio‐Rad Laboratories). Band densitometry was performed using Image Lab software (version 6.0, Bio‐Rad Laboratories), with all target proteins normalized to β‐actin loading control to account for potential variations in protein loading.

### 2.8 Statistical analysis

All statistical analyses were performed using SPSS software (version 25.0, IBM Corporation, Armonk, NY). Data are presented as mean ± standard deviation (SD) unless otherwise specified. Prior to statistical testing, data were assessed for normality using the Shapiro–Wilk test and for homogeneity of variance using Levene's test. For longitudinal data (body weight, body composition, neuromuscular function, and OGTT), a two‐way mixed‐design analysis of variance (ANOVA) was used with group (p62^−^/^−^ vs. control) as the between‐subjects factor and time as the within‐subjects factor. When significant main effects or interactions were detected, Tukey's honestly significant difference (HSD) post hoc tests were conducted to identify specific group differences. For cross‐sectional comparisons at study termination (muscle weights, myofiber CSA, and protein expression), independent samples *t*‐tests were used. The alpha level was set at *p* *<* 0.05 for all statistical tests. For multiple comparisons within the same analysis, a Bonferroni correction was applied to maintain the family‐wise error rate. All statistical tests were two‐tailed.

## 3 RESULTS

### 3.1 Food intake and body weight development

**FIGURE 1:**
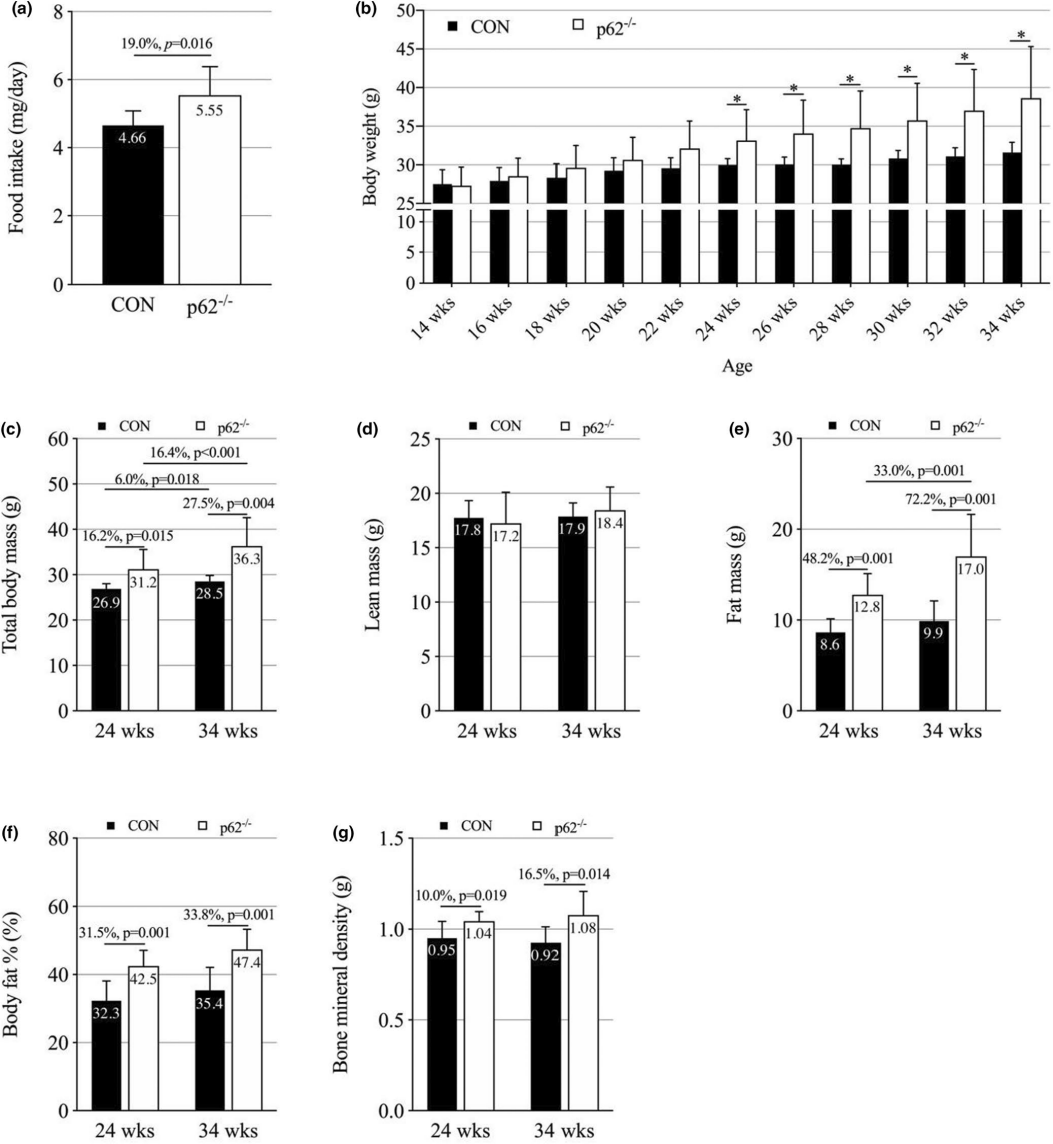
Longitudinal assessment of food intake, body weight, and body composition in p62^−^/^−^ and wild type control mice (CON). (a) Daily food intake comparison between p62^−^/^−^ and CON mice throughout the study period. (b) Body weight progression from 14 to 34 weeks of age, showing significant weight gain in p62^−^/^−^ mice starting at 24 weeks. (c) Total body mass measured by dual‐energy X‐ray absorptiometry (DXA) at 24 and 34 weeks, with p62^−^/^−^ mice displaying significantly greater total body mass at both time points. (d) Lean mass measurements showing no significant difference between groups, suggesting increased mass in p62^−^/^−^ mice is primarily due to fat accumulation. (e) Fat mass analysis showing an age‐dependent increase in adiposity in p62^−^/^−^ mice. (f) Percent body fat comparison indicating persistenctly elecated adiposity in p62^−^/^−^ mice. (g) Bone mineral density analysis revealing significantly higher BMD in p62^−^/^−^ mice at both time points. All data represent mean ± SD (*n* = 9 per group) with individual datapoints shown on bar graphs. *p < 0.05, significantly different from CON.

Throughout the 20‐week monitoring period, p62^−^/^−^ mice demonstrated significantly greater daily food intake compared to control animals (5.55 ± 0.84 g/day vs. 4.66 ± 0.42 g/day, *p* = 0.016). This represents a 19.1% increase in food consumption that remained consistent throughout the study (Figure 1a). Body weight development showed a striking divergence between groups over time, with a significant group × time interaction (*p* < 0.001). Post‐hoc analysis revealed that p62^−^/^−^ mice experienced rapid and progressive weight gain (+41.5% over 20 weeks, *p* < 0.001), while control mice showed minimal weight changes. The temporal pattern of weight gain in p62^−^/^−^ mice showed an initial period of similar growth rates until 22 weeks of age, followed by accelerated weight gain that became statistically significant from 24 weeks onward (*p* < 0.05; Figure 1b).

### 3.2 Body composition changes

Comprehensive body composition analysis revealed significant alterations in p62^−^/^−^ mice. A significant group × time interaction was observed for total body mass, with p62^−^/^−^ mice showing significantly greater total body mass at both 24 weeks (+16.2%, *p* = 0.015) and 34 weeks (+27.5%, *p* = 0.004; Figure 1c). This was primarily driven by a substantial increase in fat mass, which was significantly greater in p62^−^/^−^ mice at 24 weeks (+48.2%, *p* = 0.001) and 34 weeks (+72.2%, *p* = 0.001; Figure 1e). Consequently, percent fat mass was consistently higher in p62^−^/^−^ mice at both time points (*p* < 0.001; Figure 1f). In contrast, lean mass analysis revealed no significant main effects or interactions (*p* > 0.05; Figure 1d), indicating that the increased body mass in p62^−^/^−^ mice was primarily attributable to fat accumulation. Bone mineral density was also significantly greater in p62^−^/^−^ mice at both 24 and 34 weeks (*p* < 0.05; Figure 1g).

### 3.3 Neuromuscular function assessment

**FIGURE 2:**
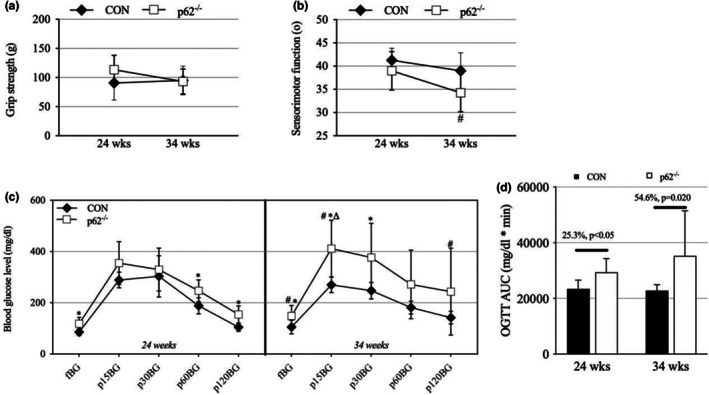
Neuromuscular function and glucose tolerance assessment in p62^−^/^−^ mice. (a) Grip strength measurements showing preserved forelimb strength in both groups across the study period, with no significant differences between groups or time points. (b) Inclined plane test assessing sensorimotor performance at 24 and 34 weeks of age. A modest decline was observed in p62^−^/^−^ mice over time, whereas control (CON) mice maintained stable performance. (c) Oral glucose tolerance test results showing fasting and post‐glucose blood glucose levels at 15, 30, 60, and 120 min. p62^−^/^−^ mice exhibited elevated glucose levels at multiple time points, indicating impaired glucose tolerance. (d) Area under the curve (AUC) analysis of the OGTT response demonstrating significantly greater glucose exposure in p62^−^/^−^ mice at both 24 and 34 weeks of age, reflecting impaired glucose clearance. All data represent mean ± SD (*n* = 9 per group). *p < 0.05, significantly different from control mice; #*p* < 0.05, significantly different from 24 weeks within the same group; Δ*p* < 0.05, significant group × time interaction.

Functional assessments revealed a mixed neuromuscular phenotype. Grip strength assessment revealed no significant main effects of time, group, or group × time interactions (*p* > 0.05), indicating that forelimb muscle strength was maintained in p62^−^/^−^ mice relative to controls (Figure 2a). However, the inclined plane test, which assesses sensorimotor function, revealed a significant main effect of time (*p* = 0.007), driven by a decline in performance in p62^−^/^−^ mice (−12.8%, *p* = 0.007), while controls showed no significant change (Figure 2b). Despite this temporal decline in p62^−^/^−^ mice, no significant between‐group differences were observed at either time point. These results suggest a selective maintenance of muscle strength but a potential impairment in more complex sensorimotor tasks.

### 3.4 Glucose tolerance assessment

Oral glucose tolerance testing revealed significant impairments in glucose homeostasis in p62^−^/^−^ mice. Fasting blood glucose (fBG) was consistently elevated in p62^−^/^−^ mice compared to controls at both 24 and 34 weeks (*p* < 0.05; Figure 2c). Following oral glucose administration, p62^−^/^−^ mice exhibited significantly elevated blood glucose levels at multiple time points (*p* < 0.05). The area under the curve (AUC) for glucose response, calculated using the trapezoidal rule, was significantly greater in p62^−^/^−^ at both 24 weeks (+25.3%, *p* < 0.05) and 34 weeks (+54.6%, *p* = 0.020; Figure 2d), confirming impaired whole‐body glucose tolerance.

### 3.5 Tissue weights and muscle morphology

**FIGURE 3:**
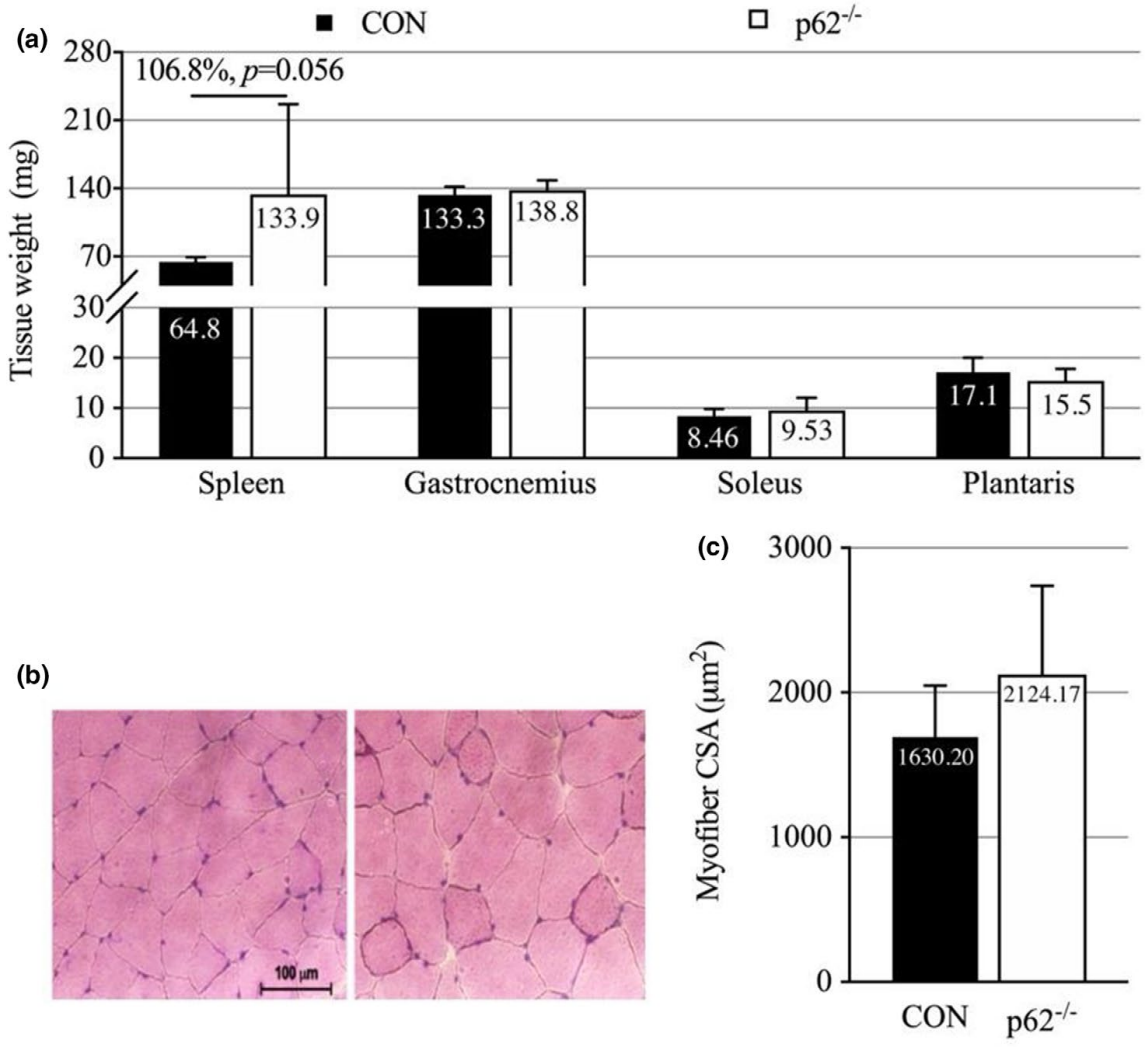
Assessment of skeletal muscle mass and morphology in p62^−^/^−^ and control mice (CON) at 34 Weeks. Panels (a)–(c) are labeled within the figure. (a) Wet tissue weights of spleen and hindlimb muscles (gastrocnemius, soleus, and plantaris). Spleen weight trended higher in p62^−^/^−^ mice, while no significant differences were found in muscle weights between groups. (b) Representative hematoxylin and eosin–stained cross‐sectional images of gastrocnemius muscle showing comparable muscle fiber morphology between CON and p62^−^/^−^ mice. Scale bar = 100 μm; magnification = 40×. (c) Quantitative analysis of myofiber cross‐sectional area (CSA) in gastrocnemius muscle revealed no significant differences between groups, indicating preserved muscle fiber size in p62^−^/^−^ mice despite obesity and metabolic dysfunction. All data represent mean ± SD (*n* = 9 per group; ≥100 fibers analyzed per animal for CSA).

At study termination, spleen weight showed a strong trend toward being increased in p62^−^/^−^ mice compared to controls (+106.8%, *p* = 0.056; Figure 3a). In contrast to the pronounced effects on adipose tissue, skeletal muscle wet weights showed no significant differences between groups. Gastrocnemius muscle weight was comparable between p62^−^/^−^ and control mice (*p* = 0.234), as were soleus (*p* = 0.456) and plantaris muscle weights (*p* = 0.378; Figure 3a). Histological analysis of myofiber cross‐sectional area (CSA) in the gastrocnemius corroborated the muscle weight findings, revealing no significant differences in fiber size between groups (*p* = 0.412; Figure 3b,c). These data indicate that muscle mass and fiber morphology were maintained in p62^−^/^−^ mice despite the presence of severe obesity.

### 3.6 Molecular analysis of muscle regulatory proteins

**FIGURE 4:**
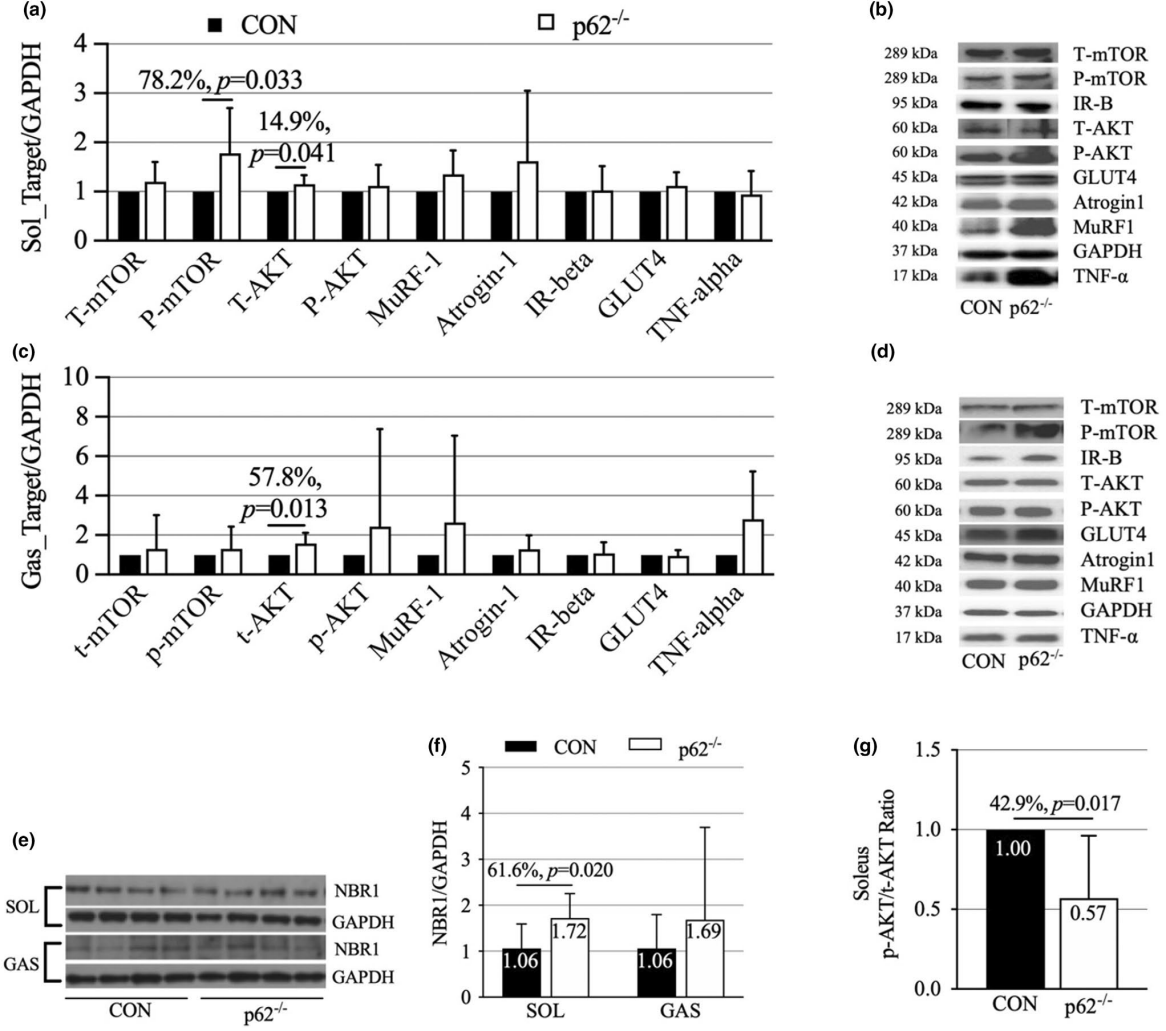
Molecular analysis of muscle regulatory proteins in p62^−^/^−^ and control mice (CON). Panels (a)–(g) are labeled within the figure. (a) Quantitative analysis of protein expression levels in soleus muscle, including total and phosphorylated mTOR (T‐mTOR and P‐mTOR), total and phosphorylated AKT (T‐AKT and P‐AKT), insulin receptor β (IR‐β), glucose transporter 4 (GLUT4), MuRF1, atrogin‐1, and tumor necrosis factor α (TNF‐α). (b) Representative western blot images of protein expression in soleus muscle. β‐actin (37 kDa) was used as a loading control. Predicted molecular weights: mTOR (289 kDa), phospho‐mTOR (289 kDa), AKT (60 kDa), phospho‐AKT (60 kDa), MuRF1 (40 kDa), Atrogin‐1 (42 kDa), IR‐β (95 kDa), GLUT4 (45 kDa), TNF‐α (17 kDa), NBR1 (130 kDa), and GAPDH (37 kDa). (c) Quantitative analysis of the same panel of proteins in gastrocnemius muscle. (d) Representative western blot images of protein expression in gastrocnemius muscle. β‐actin (37 kDa) served as loading control. (e) Representative western blot images of NBR1 in soleus and gastrocnemius muscles, a marker of autophagy dysfunction. (f) Quantitative analysis of NBR1 expression, showing significantly increased levels in soleus muscle of p62^−^/^−^ mice, suggesting impaired autophagic flux. (g) Ratio of phosphorylated to total AKT in soleus muscle, representing the activation state of this key insulin signaling protein under basal, non‐stimulated condition. All data represent mean ± SD (*n* = 9 per group). *p < 0.05, significantly different from CON.

To investigate potential mechanisms underlying the maintained muscle phenotype, we performed Western blot analysis of key regulatory proteins. In the soleus muscle, p62^−^/^−^ mice demonstrated significantly increased phosphorylated mTOR (p‐mTOR) expression (+78.2%, *p* = 0.033) compared to controls, while total mTOR levels remained unchanged, suggesting enhanced mTOR signaling (Figure 4a). Furthermore, expression of NBR1, an autophagy receptor that accumulates when autophagic degradation is compromised, was significantly increased in the soleus of p62^−^/^−^ mice (+61.6%, *p* = 0.020; Figure 4f), suggesting altered autophagic flux.

Analysis of muscle‐specific ubiquitin E3 ligases revealed no significant differences between groups in either MuRF1 or atrogin‐1 expression in both soleus and gastrocnemius muscles (all *p* > 0.05; Figure 4a,b). Similarly, the expression of key proteins in glucose uptake, insulin receptor β (IR‐β) and GLUT4, were comparable between groups in both muscle types (all *p* > 0.05; Figure 4a,b). Total AKT protein expression was significantly elevated in p62^−^/^−^ mice in both soleus (+14.9%, *p* = 0.041) and gastrocnemius muscles (+57.8%, *p* = 0.013; Figure 4a,b). There was a trend toward elevated tumor necrosis factor‐α (TNF‐α) expression in the gastrocnemius of p62^−^/^−^ mice (+179.9%, *p* = 0.057; Figure 4b), although this did not reach statistical significance.

## 4 DISCUSSION

The present investigation provides insights into the relationship between p62 deficiency, metabolic dysfunction, and skeletal muscle health at an intermediate timepoint (34 weeks). Our findings demonstrated that while p62^−^/^−^ mice developed pronounced obesity and systemic insulin resistance, skeletal muscle mass and grip strength were maintained at this stage of the disease. This phenotype is associated with molecular signatures suggesting altered mTOR and autophagy signaling. While the metabolic phenotypes of p62^−^/^−^ mice, such as obesity and glucose intolerance, have been previously reported, the present study provides the first systemic characterization of skeletal muscle mass, morphology, function, and molecular signaling in this model, representing a meaningful addition to the existing literature. The long‐term trajectory of muscle health in this model, however, remains to be determined.

The metabolic phenotype observed in our p62^−^/^−^ mice aligns with and extends previous findings demonstrating the critical role of p62 in metabolic homeostasis (Harada et al., 2013; Rodriguez et al., 2006). The significantly increased food intake (+19.1%) observed in p62^−^/^−^ mice provides a mechanistic foundation for the subsequent development of obesity. This hyperphagia likely results from central leptin resistance, as has been previously demonstrated (Harada et al., 2013; Steinberg et al., 2003). The body composition analysis clearly demonstrates that the increased body mass in p62^−^/^−^ mice is primarily attributable to fat accumulation rather than lean tissue growth, indicating that p62 deficiency specifically affects adipose tissue biology without compromising muscle tissue development in this model.

Furthermore, while extreme obesity is frequently associated with reduced bone mineral density (Cao, 2011; Núñez et al., 2007; Salamat et al., 2016), the p62^−^/^−^ mice exhibited significantly increased bone mineral density. This elevated bone mass aligns with findings in certain diet‐induced obesity models (Lecka‐Czernik et al., 2015) and may also reflect altered osteoclast activity, consistent with evidence that p62/SQSTM1 mutations strongly influence osteoclast genesis and bone remodeling (Hiruma et al., 2008; Zach et al., 2018).

Perhaps the most striking finding of our study is the maintenance of skeletal muscle mass and forelimb grip strength in p62^−^/^−^ mice despite the presence of severe obesity and systemic insulin resistance. This observation contrasts with the well‐established association between obesity and muscle dysfunction observed in both human studies (Blimkie et al., 1990; Hulens et al., 2001; Lafortuna et al., 2005) and other animal models (Griffin et al., 2010; Park et al., 2007). The maintenance of muscle wet weights, myofiber CSA, and grip strength in p62^−^/^−^ mice suggests that the mechanisms underlying muscle preservation in this model may be distinct from those operating in typical obesity scenarios.

The molecular analysis provides evidence that altered autophagy‐related signaling may be involved in the muscle phenotype of p62^−^/^−^ mice. The significantly increased expression of NBR1 (+61.6%) in the soleus muscle, an autophagy receptor that accumulates when autophagic degradation is compromised, suggests impaired autophagic flux (Salazar et al., 2019). The increased phosphorylation of mTOR (+78.2%), a master regulator of protein synthesis and an inhibitor of autophagy (Milan et al., 2015), further supports a shift in cellular signaling that could favor muscle mass maintenance, consistent with previous reports of elevated mTOR activation in the skeletal muscle of obese models (Khamzina et al., 2005; Woo et al., 2016). While these findings are suggestive, we acknowledge that these are indirect markers. Direct measurements of autophagy flux using LC3‐II turnover assays or lysosomal inhibitors would be needed to definitively establish autophagy suppression. Additionally, the accumulation of NBR1 could reflect compensatory upregulation rather than impaired clearance.

Several alternative or complementary mechanisms may contribute to muscle preservation in p62^−^/^−^ mice. First, the chronic mechanical loading from increased body weight may provide an anabolic stimulus that counteracts metabolic stress (Ehrlich & Lanyon, 2002). Second, the absence of significant changes in muscle‐specific E3 ubiquitin ligases (MuRF1 and atrogin‐1) suggests that the proteasome‐mediated protein degradation pathway is not activated. Third, potential alterations in mitochondrial function, inflammatory signaling, or satellite cell activity could influence muscle homeostasis. Future studies should systematically evaluate these possibilities to provide a comprehensive mechanistic understanding. Additionally, the trend toward elevated TNF‐α expression in the gastrocnemius of p62^−^/^−^ mice is consistent with its well‐characterized role as an inflammatory mediator linking obesity to insulin resistance (Hotamisligil, 1999; Kern et al., 1995).

### 4.1 Study limitations and future directions

Several important limitations should be acknowledged. First, our assessment of autophagy relied on indirect markers (NBR1 and p‐mTOR). Direct measurements of autophagy flux such as LC‐2 turnover in the presence of lysosomal inhibitors would be necessary to confirm impaired autophagy. Second, we did not quantify rates of protein synthesis or degradation rates, which are essential for understanding the net protein balance governing muscle maintenance (Castillero et al., 2013; Foletta et al., 2011). Third, all Western blot analyses were performed under basal, resting conditions. Notably, insulin signaling was assessed without an insulin stimulation challenge; therefore, the absence of differences in basal phospho‐AKT does not exclude potential deficits in insulin responsiveness in p62^−^/^−^ muscle. Future studies should assess insulin‐stimulated signaling to better define metabolic sensitivity. Fourth, although total GLUT4 protein abundance was measured, its translocation to the plasma membrane was not assessed. Fifth, while intramuscular TNF‐α was determined, systemic inflammatory status remains unclear, as plasma cytokines and additional mediators (e.g., NFκB and IL‐6) were not assessed. Sixth, functional assessments were limited to grip strength and the inclined plane test. More comprehensive assessments, including treadmill performance or ex vivo muscle contractility, would provide a fuller picture of neuromuscular function. Finally, this study included only male mice over a 20‐week period. Long‐term effects of p62 deficiency and potential sex differences warrant further investigation.

Future research should address these gaps through (1) direct quantification of protein synthesis and degradation rates to validate shift in protein balance, (2) assessment of mitochondrial function and biogenesis to determine preservation of metabolic capacity, and (3) investigation of muscle stem cell activity and regenerative potential to evaluate long‐term sustainability of muscle integrity in p62^−^/^−^ mice.

## 5 CONCLUSION

This investigation of p62‐deficient male mice reveals a metabolic phenotype characterized by the coexistence of severe obesity and systemic insulin resistance with maintained skeletal muscle mass and grip strength at 34 weeks of age. The key findings demonstrate that p62^−^/^−^ mice develop progressive obesity through increased food intake and enhanced adipogenesis, leading to significant metabolic dysfunction. Despite these metabolic impairments, skeletal muscle tissue is preserved in terms of mass, morphology, and forelimb strength at these intermediate time points. These findings likely reflect the initial phase of the obesity‐muscle relationship, where increased body mass and altered metabolic signaling have not yet produced overt muscle pathology. In the longer term, the sustained inflammatory and metabolic burden of severe obesity would be expected to negatively impact skeletal muscle.

The molecular analysis suggests that this muscle‐sparing phenotype is associated with upregulated mTOR signaling and altered autophagy receptor expression (NBR1). The p62^−^/^−^ mouse model represents a valuable tool for studying the complex relationships between autophagy, metabolism, and muscle homeostasis. Future research should include longitudinal studies extending beyond 34 weeks to determine whether the apparent preservation of muscle mass is sustained over time. Additional work should also incorporate direct measurements of protein synthesis and degradation rates, assessment of autophagic flux using established biochemical methods, and evaluation of insulin‐stimulated signaling to more fully characterize metabolic impairment in skeletal muscle. Furthermore, inclusion of markers of mitochondrial content and function (e.g., PGC‐1α, citrate synthase activity, and OXPHOS subunit proteins) would help assess the bioenergetic status of skeletal muscle in this model.

## AUTHOR CONTRIBUTIONS

**Do‐Houn Kim:** Conceptualization; data curation; formal analysis; funding acquisition; investigation; methodology; project administration; resources; software; validation; visualization. **Gloria Salazar:** Methodology; resources; supervision. **Alex Klemp:** Data curation; methodology. **Hyun‐Seok Hwang:** Data curation; methodology; resources. **Mingchia Yeh:** Data curation; validation; visualization. **Lynn B. Panton:** Conceptualization; supervision. **Jeong‐Su Kim:** Conceptualization; funding acquisition; investigation; methodology; supervision.

## FUNDING INFORMATION

This research was financially supported by Jiwon Co. (South Korea) and the dissertation grant provided by the College of Human Sciences at the Florida State University. The funding bodies had no role in the design of the study, collection, analysis, and interpretation of data, or in writing the manuscript.

## CONFLICT OF INTEREST STATEMENT

The authors declare that they have no competing interests related to this work.

## ETHICS STATEMENT

All experimental procedures were conducted in strict accordance with the “Principles of Laboratory Animal Care” (NIH publication No. 85–23, revised 1985) and were approved by the Animal Care and Use Committee of the Florida State University (Protocol number: 1703). No human subjects were involved in this study.
